# Long-term effect of hand-arm vibration on thermotactile perception thresholds

**DOI:** 10.1186/s12995-018-0201-1

**Published:** 2018-06-22

**Authors:** Ronnie Lundström, Adnan Noor Baloch, Mats Hagberg, Tohr Nilsson, Lars Gerhardsson

**Affiliations:** 1 Department of Radiation Sciences, Umeå University, SE-901 87 Umeå, Sweden; 2 Department of Occupational and Environmental Medicine, University of Gothenburg and Sahlgrenska University Hospital, Gothenburg, Sweden; 3 Department of Occupational and Environmental Medicine, Umeå University, SE-901 87 Umeå, Sweden

**Keywords:** Hand-arm vibration, Hand-transmitted vibration, Hand, Thermotactile perception

## Abstract

**Background:**

Occupational exposure to hand-transmitted vibration (HTV) is known to cause neurological symptoms such as numbness, reduced manual dexterity, grip strength and sensory perception. The purpose of this longitudinal study was to compare thermotactile perception thresholds for cold (TPT_C_) and warmth (TPT_W_) among vibration exposed manual workers and unexposed white collar workers during a follow-up period of 16 years to elucidate if long-term vibration exposure is related to a change in TPT over time.

**Methods:**

The study group consisted of male workers at a production workshop at which some of them were exposed to HTV. They were investigated in 1992 and followed-up in 2008. All participants were physically examined and performed TPT bilaterally at the middle and distal phalanges of the second finger. Two different vibration exposure dosages were calculated for each individual, i.e. the individual cumulative lifetime dose (mh/s^2^) or a lifetime 8-h equivalent daily exposure (m/s^2^).

**Results:**

A significant mean threshold difference was found for all subjects of about 4–5 °C and 1–2 °C in TPT_W_ and TPT_C_, respectively, between follow-up and baseline. No significant mean difference in TPT_C_ between vibration exposed and non-exposed workers at each occasion could be stated to exist. For TPT_W_ a small but significant difference was found for the right index finger only. Age was strongly related to thermotactile perception threshold. The 8-h equivalent exposure level (A (8)) dropped from about 1.3 m/s^2^ in 1992 to about 0.7 m/s^2^ in 2008.

**Conclusions:**

A lifetime 8-h equivalent daily exposure to hand-transmitted vibration less than 1.3 m/s^2^ does not have a significant effect on thermotactile perception. Age, however, has a significant impact on the change of temperature perception thresholds why this covariate has to be considered when using TPT as a tool for health screening.

## Background

Hand-transmitted vibration (HTV) may lead to neurological, vascular, and musculoskeletal disorders in the upper extremity. The symptoms, that may occur singly or in different combinations, are collectively denoted as the hand-arm vibration syndrome (HAVS) [1]. The neurological component of HAVS is characterized by diffusely distributed peripheral neuropathy with predominant symptoms of sensory impairment, The most common symptoms are subjective experience of digital paraesthesia and numbness, deterioration of sensory perception (i.e. vibration, cold, warmth, pain), and loss of manipulative dexterity [2, 3].

Hand intensive work, including exposure to HTV, is associated with an increased risk of impaired thermal perception (eg. [4–7]). Interestingly, exposure to vibration seems to affect perception of cold more compared with warmth [8, 9]. Moreover, an exposure-response relationship between HTV and thermal perception has been suggested in some studies (eg. [5, 10]. For vibration-induced thermotactile impairment the conceivable target structures are the end organs, the thinly myelinated (A-delta), and the small calibre non-myelinated (C) fibres [11]. Experiments addressing temporary thermotactile threshold shift induced by vibration indicate an effect, especially on cold compared with warmth (eg. [8, 12]). Hypoaesthesia of the sensation of warmth is claimed to be more prevalent at the early stages of vibration disease whereas hypoesthesia to cold occurs at more advanced stages of hand-arm vibration disease [9]. The diversity of symptoms expressed by long-term vibration exposed workers implies that different pathophysiological mechanisms may affect the degeneration of small fibre neuropathy [13]. Some workers may develop quite severe neurophysiological symptoms and signs within a few years, while others with similar exposure for decades develop no or only minor disturbances. The reason for this is still unclear. The prevalence of peripheral sensorineural disorders among vibration-exposed workers varies from a few per cent to more than 80% [14]. The awareness of the importance as well as relatively high prevalence of sensory neuropathy has entailed an increasing interest to get a deeper knowledge of the causes of small fibre neuropathy as well as the underlying pathophysiological mechanisms. Quantitative sensory testing is a psychophysical neurological test battery that can examine subgroup changes in different nerve fibre functions, mainly linked to A-delta and C-nerve fibres, and thus useful for screening and diagnosis of vibration induced neuropathy (eg. [2, 3, 15–17]).

The aim of the present longitudinal study is to explore whether a long-term occupational exposure to HTV lead to a deterioration of the thermotactile sense among a group of workers employed at a heavy production workshop.

## Methods

### Study group

This longitudinal study is based on a sample from a cohort consisting of male white- and blue-collar workers at a plant that produces heavy equipment for paper and pulp mills that was investigated in 1992 (*n* = 229) and followed-up in 2008 (*n* = 228). At both occasions basic information about age, work assignment, years at work, general state of health, previous and present exposure to vibration and more was collected in a questionnaire. All participants were physically examined by one and same occupational physician. The inclusion criteria for this study were; 1) Participation at both or any occasions with thermotactile perception threshold (TPT) measurements on the volar side of the index finger, and 2) Not having symptoms of diseases known to cause sensory neuropathies, such as diabetes, metabolic disturbances, and carpal tunnel syndrome. For more information about the criteria for inclusion, see Nilsson and Lundström 2001 [5]. At baseline (1992) 140 study (out of 229) participants had TPT measurements, and at follow-up (2008) 142 study participants (out of 228) had TPT measurements. Among these, 119 study participants had TPT measurements at both occasions, 21 only at the baseline (1992) and 23 only at follow-up. Our study is approved by the Regional Ethics Review Board at Umeå University (Registration number 97–76 and 2007-161 M) and conducted accordingly. All subjects signed an informed consent before entering the study.

### Thermotactile perception thresholds

Thermotactile perception thresholds for cold (TPT_C_) and warmth (TPT_W_) was at both occasions in 1992 and 2008 determined using a modification of the Marstock method [18] with computer assisted automatic exposure and response recording (Thermotest; Somedic, Sales AB, Sweden). A thermostimulator, i.e. a Peltier element-based contact thermode (25 × 50 mm), was applied to the skin on the volar surface of the two distal phalanges (i.e. the middle and distal phalanx) of the second digit (lengthways along the finger). The TPT_C_ and TPT_W_ induced by contact temperature were assessed by the method of limits. The rate of the temperature change was linear and about 1 °C/s. For TPT measurement conducted 1992 the skin temperature, measured by contact thermometry, was used as the start temperature. In this way a neutral starting temperature was accomplished that was perceived as indifferent, i.e. nor warm nor cold. For measurement conducted 2008 the start temperature was however fixed and set to 32 °C. The subject was instructed to press a button on a hand switch when a sensation of warmth or cold was perceived. The operating temperature range was set to 10–52 °C. After the subject’s response the temperature of the thermostimulator returned to the pre-set starting temperature. The measurement of warmth and cold was repeated 10 times. The threshold was taken as the mean of the measurements. The interstimulus interval for all threshold measurements was randomly distributed within 2 s.

### Assessment of vibration exposure

Personal vibration doses were estimated 1992 and 2008 for all exposed workers through measurement on all types of tools used at all relevant job stations. Hand transmitted vibration most often occurred from use of grinders that was used for grinding, polishing, and cutting. Hammers and nut wrenches were used for finishing welding seams and assembly of machinery. The vibration magnitudes, in terms of frequency-weighted acceleration level (SI: m/s^2^), were measured in accordance with the international standard ISO 5349–1 [19]. A detailed description of measurement conducted in 1992 is reported elsewhere [20]. Measurement conducted in 2008 was done accordingly. The daily duration of exposure to vibration for each individual was estimated through observation at the workstation. The observer noted the kind of tool the operator was handling, whether the machine was working, and which hand that was exposed during an observation time of 150 min. Furthermore, all workers were interviewed in order to obtain information about their entire lifetime exposures, about the number of years in different work, types of exposure, and duration of exposure per day. On that basis two different lifetime vibration doses (LTVD1 and LTVD2) was calculated for each individual worker using the following formulas;

$$ \mathrm{LTVD}1=\sum \limits_i^n{a}_{wi}{t}_{Ti} $$

(mh/s^2^), and

$$ \mathrm{LTVD}2\kern0.5em =\kern0.5em {\mathrm{A}}_{\mathrm{w}(8)}={\left(\Sigma \left({{\mathrm{a}}_{\mathrm{w}\mathrm{i}}}^2\cdot {\mathrm{t}}_{\mathrm{i}}\right)/\left(60.{\mathrm{T}}_{(8)}\right)\right)}^{1/2} $$

(m/s^2^) where; *a*_*wi*_ is the frequency weighted acceleration level for vibrating tool *i*, *t*_*i*_ is the exposure time for tool *i*, and *t*_*Ti*_ the total lifetime exposure (i.e. hours/workday · workdays/year · years; workdays/year was set to 200) for tool *i*.

LTVD1 thus reflects an individual’s cumulative lifetime dose based on the total number of hours with vibration exposure where as LTVD2 reflects a lifetime 8-h equivalent daily exposure. For more information, see [21].

The study group (ALL) was dichotomized in to sub-groups, i.e. exposed to HTV (EHTV) and not exposed to HTV (NEHTV). For statistical calculation based on LTDV1 and LTVD2, EHTV is defined as those workers having LTDV1 > 1600 mh/s^2^ and LTDV2 > 0.5 m/s^2^, respectively. The rationale for the LTDV1 dichotomization is discussed elsewhere [20]. The dichotomization level for LTDV2 was set to one fifth of the daily exposure action value of 2.5 m/s^2^ specified in the current EU Directive [22].

### Statistical analysis

Descriptive statistics of variables of interest was performed separately for exposed and referents at both time point, i.e. 1992 and 2008. We used longitudinal regression models to investigate the relationship between the outcomes and explanatory variables. By using SAS procedure PROC MIXED one can use all available data in analysis instead of ignoring subjects with missing data**,** hence having more statistical power [23–25]. We have used PROC MIXED and used all available data in our analysis, which resulted in different number of data points (subjects) in tables for descriptive statistics at baseline and follow-up. We assumed fixed effects, i.e. the model holds true across the sample and with the same slope. In other words, exposure will affect all persons in the same way. These models study both between- and within-subject changes over time.

First simple longitudinal regression models were used to investigate the relationship between one outcome and one predictor variable at a time. Finally multivariate longitudinal regression models were built partly based on the results of simple regression analyses and partly on the researchers clinical experience. The longitudinal regression analyses yielded beta values (regression coefficients) with standard error and *p*-values. It also yielded least square means (LSM) and differences in LSM to compare Categorical variable’s means adjusted for other variables and averaged across the repeated measures. Statistical significance, alpha was set at 0.05. All statistical analyses were performed with SAS 9.4 for windows (SAS Institute Inc., Cary, NC, USA).

## Results

Descriptive statistics for age, height, temperature perception thresholds and vibration dosages for all included workers as well as for the two sub-groups, i.e. EHTV and NEHTV, are presented in Table 1. At baseline and follow-up, there was a mean difference of 3.7 years (CI 95%; 0.1–7.4) and 5.6 years (CI 95%; 1.8–9.3) for the two sub-groups. There was no significant difference in height between the two groups at baseline and at follow-up.

**Table 1:** Descriptive statistics for study group and its sub-groups during the follow-up period 1992–2008. Mean thermotactile perception thresholds for cold (TPT_C_) and warmth (TPT_W_) measured on the volar side of two distal phalanges on the right and left index finger among vibration exposed (ETHV) and un-exposed (NETHV) workers during the follow-up period. LTVD1 and LTVD2 are two different lifetime vibration doses. For more information, see text

	1992			2008		
	ALL (*n* = 140)	NEHTV (*n* = 41)	EHTV (*n* = 99)	ALL (*n* = 142)	NEHTV (*n* = 35)	EHTV (*n* = 107)
	Mean (95% CI)	Mean (95% CI)	Mean (95% CI)	Mean (95% CI)	Mean (95% CI)	Mean (95% CI)
Age (years)	41 (39, 42)	43 (40, 46)	39 (38, 41)	55 (53, 57)	59 (56, 62)	54 (52, 56)
Height (cm)	179 (178, 180)	179 (178, 181)	179 (178, 180)	179 (178, 180)	179 (177, 181)	179 (178, 180)
LTVD1 (mh/s^2^)× 10^3^	21.0 (16.7, 25.3)	0	29.7 (24.5, 35.0)	28.4 (23.7, 33.2)	0.0 (0, 0.0)	37.7 (32.6, 42.9)
LTVD2 (m/s^2^)	0.9 (0.7, 1.2)	0	1.3 (1.1, 1.6)	0.6 (0.4, 0.7)	0	0.7 (0.6, 0.9)
Right hand						
TPT_W_ (°C)	34.1 (33.6, 34.6)	33.1 (32.5, 33.7)	34.5 (33.8, 35.2)	38.8 (38.3, 39.4)	38.5 (37.5, 39.6)	38.9 (38.3, 39.6)
TPT_C_ (°C)	25.0 (24.4, 25.5)	25.3 (24.3, 26.2)	24.9 (24.2, 25.5)	26.6 (26.0, 27.2)	25.7 (24.1, 27.3)	26.9 (26.3, 27.5)
Left hand						
TPT_W_ (°C)	33.7 (33.2, 34.2)	33.2 (32.5, 34.0)	33.9 (33.2, 34.5)	38.4 (37.9, 38.9)	38.3 (37.1, 39.4)	38.5 (37.8, 39.1)
TPT_C_ (°C)	25.2 (24.6, 25.8)	25.7 (24.8, 26.7)	25.0 (24.2, 25.7)	26.9 (26.2, 27.5)	26.6 (24.8, 28.5)	27.0 (26.3, 27.6)

Simple longitudinal regression analyses showed that all predictor variables had a significant relationship with all temperature perception thresholds. We investigated the following predictor variables; age, height, LTVD1, LTVD2, ELTVD1 (binary exposure variables based on LTVD1), ELTVD2 (binary exposure variables based on LTVD2) and Year (1992 and 2008) (Table 2).

**Table 2:** Univariate analyses of thermotactile perception thresholds for cold (TPT_C_) and warmth (TPT_W_) measured on the volar side of the two distal phalanges on the right and left index finger with four explanatory variables, vibration exposure (LTVD1 and LTVD2), age and height

	Left index finger				Right index finger			
	TPT_C_		TPT_W_		TPT_C_		TPT_W_	
	β	SE	β	SE	β	SE	β	SE
Age	.5	0.01	0.7	0.001	0.5	0.01	0.7	0.01
Height	.1	0.002	0.2	0.001	0.1	0.001	0.2	0.001
LTVD1	.0002	0.00003	0.0004	0.00004	0.0002	0.00003	0.0005	0.00004
LTVD2	−0.6	0.2	−1.0	0.4	−2.0	0.2	1.1	0.3
ELTVD1								
(Exp)	26.1	0.3	36.3	0.3	26.0	0.3	36.8	0.25
(Not-Exp)	26.0	0.5	35.6	0.4	25.4	0.5	35.6	0.42
Mean difference	0.04 (0.6)		0.7 (0.5)		0.5 (0.5)		**1.2 (0.5)**	
ELTVD2								
(Exp)	25.5	0.4	36.0	0.4	25.3	0.4	36.2	0.37
(Not-Exp)	26.4	0.3	36.1	0.3	26.1	0.3	36.6	0.28
Mean difference	−0.9 (0.5)		−0.1 (0.5)		−0.8 (0.4)		−0.4 (0.5)	
Year								
(1992)	25.2	0.3	33.7	0.3	25.1	0.3	34.1	0.27
(2008)	26.9	0.3	38.4	0.3	26.6	0.3	38.8	0.27
Mean difference	**−1.7 (0.3)**		**−4.7 (0.3)**		**−1.5 (0.3)**		**−4.7 (0.3)**	

Significant differences in bold

For left index finger, vibration exposed workers (ELTVD1) had a mean level of 26.1 °C and non-vibration exposed workers had a mean level of 26.0 °C for TPT_C_ during the follow-up period. The difference in these means was not significant, see column 1 in Table 2. There were not any significant mean difference in TPT_C_ and TPT_W_ (left index finger) between vibration exposed and non-exposed workers based on LTVD1 and LTVD2, respectively. For TPT_W_ on the right index finger a small but significant difference was however found.

As can be seen in Table 2 there was a significant mean difference in TPT_C_ and TPT_W_ for both fingers between the two occasions. As an example, the mean TPT_C_ for left index finger among all workers in 1992 was 25.2 °C compared to 26.9 °C in 2008, i.e. a mean difference of about 1.7 °C. Corresponding figures for TPT_W_ was 33.7 °C and 38.4 °C, i.e. a mean difference of about 4.7 °C. Similar figures is valid for the right index finger. This means that subjects need more heat stimuli and less cold stimuli for thermotactile perception at follow-up.

To further elucidate the influence of vibration exposure on the temperature perception thresholds, multivariate analyses were performed with TPT_C_ and TPT_W_ as outcome variables. We built four multivariate models with one exposure variable adjusted for age and height for each outcome (Table 3). The model including the dichotomous exposure variable ELTVD1 based on LTVD1 and adjusting for age and height resulted in similar beta-coefficients for exposed and not-exposed workers for all TPT-indices (Table 3). Comparison of LSM reveals significant differences between exposed and not exposed for TPT_W_ but not for TPT_C_. These differences were between 1.5–2.0 °C. A similar result was noted when using dichotomous exposure variable ELTVD2 based on LTVD2 and adjusting for Age and Height in the models.

**Table 3:** Results from longitudinal regression analysis of four multivariate models (Model 1 to 4). Thermotactile perception thresholds for cold (TPT_C_) and warmth (TPT_W_) are outcome variables, and vibration doses (LTVD1 and LTVD2, respectively), age and height are explanatory variables. All available data is included in the analysis. For more information, see text

	Left index finger						Right index finger					
	TPT_C_			TPT_W_			TPT_C_			TPT_W_		
Model 1	ß (SE)	*p*	LSM	ß (SE)	*p*	LSM	ß (SE)	*p*	LSM	ß (SE)	*p*	LSM
LTVD1	−0.00001 (0.00001)	.25		0.00002 (0.00001)	.027		0.000007 (0.00001)	.5		0.00002 (0.00001)	.04	
Age	0.07 (0.02)	<.0001		0.2 (0.02)	<.0001		0.04 (0.02)	.01		0.19 (0.017)	<.0001	
Height	0.13 (0.004)	<.0001		0.1 (0.004)	<.0001		0.1 (0.004)	<.0001		0.15 (0.005)	<.0001	
Model 2												
LTVD1 Exp	23.6 (8.3)	.02	26.1	14.9 (7.4)	.003	**36.5**	26.1 (7.4)	.001	26.0	15.7 (6.6)	<.0001	**37.0**
LTVD1 NExp	23.4 (8.3)	.02	25.9	13.4 (7.4)	.003	**35.0**	25.4 (7.4)	.001	25.4	13.7 (6.6)	<.0001	**35.0**
Age	0.1 (0.02)	<.001		0.2 (0.02)	<.0001		0.03 (0.02)	.05		0.2 (0.02)	<.0001	
Height	−0.002 (0.05)	.97		0.1 (0.04)	0.1		−0.01 (0.04)	.8		0.1 (0.04)	.1	
Model 3												
LTVD2	−0.2 (0.2)	.41		0.7 (0.2)	.003		−0.5 (0.2)	.03		0.8 (0.21)	.0003	
Age	0.1 (0.02)	< .001		0.2 (0.02)	<.0001		0.03 (0.02)	.08		0.2 (0.02)	<.0001	
Height	0.1 (0.01)	<.0001		0.1 (0.01)	<.0001		0.1 (0.01)	<.0001		0.1 (0.01)	<.0001	
Model 4												
LTVD2 Exp	23.9 (8.3)	.02	25.7	15.1 (7.3)	.004	**37.0**	26.4 (7.4)	.004	25.4	15.3 (6.8)	.002	**37.5**
LTVD2 NExp	24.5 (8.3)	.02	26.3	13.6 (7.3)	.004	**35.5**	27.0 (7.4)	.004	26.1	13.7 (6.8)	.002	**35.9**
Age	0.1 (0.02)	<.002		0.2 (0.02)	<.0001		0.02 (0.02)	.2		0.2 (0.02)	<.0001	
Height	−0.004 (0.05)	.94		0.1 (0.04)			−0.01 (0.04)	.8		0.1 (0.04	.07	

Significant differences between LSM in bold

When adjusting the vibration doses LTVD1 and LTVD2 for age and height in Model 1 and 3, respectively, the association between vibration doses and TPT_C_ disappeared. However, we observed a significant association between vibration doses and TPT_W_ even after adjusting for age and height but with a smaller magnitude.

As an example, from Model 1 for TPT_W_ of right index finger we have the following regression equation;

1 $$ {\mathrm{TPT}}_{\mathrm{W}}=1.7\times {10}^{\hbox{-} 5}\left(\mathrm{mh}/{\mathrm{s}}^2\right)+0.2\times \mathrm{Age}+0.2\times \mathrm{Height} $$

This means that the thermotactile threshold for warmth (TPT_W_) is estimated to increase by 1.7 °C for every 100,000 h of vibration exposure (i.e. 12,500 days with 8 h daily exposure) if the effect of age and height was kept constant.

## Discussion

A significant difference in thermotactile perception thresholds when comparing baseline and follow-up has been found. For TPT_W_ a small but significant difference was found for the right index finger only (unadjusted). Significant differences in thermotactile perception thresholds for warmth were found for both hands after adjusting for age and height. However, no significant differences in this respect were found for cold. The majority of the workers (96% in 1997), were right-handed. Smaller and lighter vibrating tools are usually held in the dominant hand, in this case mainly in the right hand. Larger and heavier vibrating tools are usually held in both hands. Thus, the right hand will have a higher vibration exposure than the left hand in right handed workers. Accordingly, the right hand will have a lower temperature perception threshold for cold and a higher temperature perception threshold for warmth than the left hand as shown in our study. Age, however, had a strong impact on the change of temperature perception thresholds and is therefore an important covariate in this context.

The vibration exposure has decreased during the follow-up period as shown in Table 1. This is supported by the fact that the mean current life-time 8-h equivalent exposure level (A (8)), that was about 1.3 m/s^2^ among the workers in 1992 dropped to about 0.7 m/s^2^ in 2008. The main reasons for the reduction of vibration exposure are technical preventive measures (e.g. usage of isolation gloves and less vibrating tools), improved medical surveillance and the replacement of manual tasks with robotic controlled processes. A reason for not finding a significant impact from the vibration exposure at follow-up may thus be that the general exposure after 1992 was reduced to a level considerably lower than the action level of 2.5 m/s^2^ specified in EU:s health and safety directive [22].

It is clear that the magnitude of an individual’s thermotactile threshold is depending on the starting temperature of the test. In 1992 the starting temperature was adjusted to the individuals actual skin temperature. In 2008, however, a fixed starting temperature of 32 °C was used. This shift in methodology is of course not optimal but was due to a modification of the standardized protocol for TPT measurements in our country. We know that the thermotactile sense is sensitive to a sudden change in temperature within a relatively narrow range, approximately less than ±3–4 °C, and more or less independent of starting point. So, an individual’s absolute TPT level will thus differ if a test and re-test TPT measure is taken using different starting temperatures. A starting temperature of 29 °C in 1992 would thus yield an approximate TPT span between 25 °C and up to 33 °C for cold and warmth, respectively. A corresponding span from a starting temperature of 32 °C would then be approximately 28 °C to 36 °C. Due to this methodological difference the absolute TPT values measured in 1992 and 2008 cannot be directly compared. As it now looks in Table 1, the sensitivity to cold had improved during the follow-up period while the sensitivity to warmth had deteriorated. This pattern is common for both sub-groups, i.e. for vibration-exposed workers as well as for non-vibration exposed workers. This does not affect our findings when comparing TPT values between vibration-exposed workers with non-vibration exposed workers.

In Table 3, the β (regression)-coefficients for the explanatory variables with temperature perception thresholds as outcome variables are listed. If assuming a mean vibration exposure dose of 8000 mh/s^2^ during the follow-up period (Table 1) an increase of 0.14 degrees of the warmth threshold in dig 2 right hand would be expected while keeping the effect of age and height constant (Table 3). Using the mean values from the descriptive statistics in Table 1 in regression eq. (1), we will have a LSM of 35.1 °C at baseline and 37.9 °C at follow-up, with an expected increase of 2.8 °C for the ALL group. This is a fair estimate of the real difference of 4.7 °C shown in Table 2. As seen in this example, a length of + 10 cm from 180 cm to 190 cm would give an increase of TPT_W_ of 1.5 °C (Table 3) while keeping the vibration dose and age constant. An explanation for this effect is that a longer peripheral nerve pathway also led to a longer transmission time from end organ to cortex. This extra transmission time enables the continuously increasing or decreasing stimulus to increase a little bit further before it is perceived. Also in other studies, age has shown an impact on thermal thresholds of quantitative sensory testing [26–31]. In a study of 484 normal subjects, Lin and co-workers [27] found that age was consistently and significantly correlated with sensory thresholds of all tested modalities and had a stronger impact on the multivariate model compared to other factors such as gender, body height, body weight and body mass index. The authors concluded that age had the strongest impact on sensory thresholds compared with other factors of gender and anthropometric parameters. Separate tests are recommended for cold and warm determinations and these measurements should not be replaced by a single measurement such as the neutral-zone gap [5, 16]. On the contrary, Seah and Griffin [32] show a small and insignificant effect and conclude that an age correction may not be needed for persons aged between 20 to 65 years.

Not only the size but also the position of the finger on the thermode can influence the level of TPT. In this study we have chosen to measure perception with the two distal phalanxes of one finger in contact with the thermode in order to cover the major part of the stimulus area. It can be questioned whether this is a good or bad arrangement. In this longitudinal study we have used the same thermode and methodological arrangement at both occasions that enables direct comparisons.

One problem with longitudinal studies with follow-up periods of 10 to 15 years or longer is that it is difficult to use the same equipment during the whole study period. The measuring equipment is “aging” and may need to be replaced. Sometimes it also becomes increasingly difficult to find spare parts. Even if the original equipment is available it might not be comparable to the one that was used 15–20 years ago. During such a long study period there will also be improved technological changes that may be desirable to use. It can be difficult to compare the new equipment with the measures from the old one. These problems are growing with the length of the study period. In this study the measuring equipment has however been basically the same both at baseline and at follow-up.

Moreover, no generally accepted reference materials for the determination of temperature perception thresholds are available when the thermometry equipment is bought. This means that all users will have to collect their own reference values for TPT cold and warmth, respectively. Accordingly there may be some differences in reference values when comparing different research centres in different parts of the world. We have collected a reference sample with a normal range between 23 and 42 °C for male subjects less or equal to 44 years and between 20 and 45 °C for subjects 45 years and older. There seems to be a breaking point around 45 years of age. After that point we can see a slight deterioration of the neurosensori sensitivity. If using these criteria almost all subjects in the study group and in the reference group will fall within the normal range in this study. Hafner and co-workers found a clear effect of age on thermal thresholds in a study of 101 normal volunteers but no significant effect on gender was noted [26]. They also found some differences between the three operators that performed the testing.

It is also important that the examiner is experienced with the test and can understand and respond if the test subject doesn’t understand the instructions or is conducting the testing in an improper way. At both occasions in this study the test were performed by a qualified examiner.

Another factor that must be considered is the thickness of the nerve fibres. A-delta fibres are thicker than the C-fibres giving an estimated velocity of 12–30 m/s versus 0.5–2 m/s, which may affect the response time. Also, the cognitive set of the subject may influence the response to cold or warm stimuli. The subjects are asked to respond when they feel a temperature shift from neutral to cold or from neutral to warm. A careful and meticulous person may wait a little longer with the response compared to a subject with another type of personality. In this study, however, all workers and referents were their own controls, investigated the same way in 1992 and in 2008. Thus, we don’t think that any of these facts would have influenced the final results.

## Conclusions

A lifetime 8-h equivalent daily exposure to hand-transmitted vibration less than 1.3 m/s^2^ does not have a significant effect on thermotactile perception. Age, however, has a significant impact on the change of temperature perception thresholds why this covariate has to be considered when using TPT as a tool for diagnosis or health screening.

## Abbreviations

ALL: All individuals in the study group
EHTV: Individuals exposed to hand-transmitted vibration
ELTVD1: Binary exposure variables based on LTVD1
ELTVD2: Binary exposure variables based on LTVD2
HAVS: Hand-arm vibration syndrome
HTV: Hand-transmitted vibration
LSM: Least square means
LTVD1: Reflects an individual’s cumulative lifetime dose based on the total number of hours with vibration exposure
LTVD2: Reflects an individual’s lifetime 8-h equivalent daily vibration exposure
NEHTV: Individuals not exposed to hand-transmitted vibration
TPT: Thermotactile perception threshold
TPT_C_: Thermotactile persception threshold for cold
TPT_W_: Thermotactile perception threshold for warmth
